# Treatment-Based Recurrence of Scar Pregnancy: A Systematic Review of the Literature as a Tool for More Informed Decision-Making

**DOI:** 10.3390/jcm15166239

**Published:** 2026-08-12

**Authors:** Stefania Carlucci, Laura Vona, Teresa Loverre, Antongiulio Capobianco, Alessandro Favilli, Guglielmo Stabile, Stefano Bettocchi

**Affiliations:** 1 Department of Clinical and Experimental Medicine, Institute of Obstetrics and Gynecology, University of Foggia, 71122 Foggia, Italy; s.carlucci86@gmail.com (S.C.); loverretitti@gmail.com (T.L.); antongiulio.capobianco@unifg.it (A.C.); stefano.bettocchi@unifg.it (S.B.); 2 Department of Medicine and Surgery, Section of Obstetrics and Gynecology, University of Perugia, 06135 Perugia, Italy; alessandro.favilli@unipg.it; 3 Department of Medical and Surgical Sciences, Institute of Obstetrics and Gynecology, University of Foggia, 71122 Foggia, Italy; guglielmost@gmail.com

**Keywords:** cesarean scar pregnancy, recurrent cesarean scar pregnancy, recurrence, treatment, reproductive outcomes

## Abstract

**Objectives**: Caesarean scar pregnancy (CSP) is a rare but potentially life-threatening ectopic pregnancy whose incidence has increased in recent years. Although several surgical and non-surgical treatments have been proposed, outcomes remain heterogeneous. This systematic review aimed to compare recurrence rates of CSP according to different treatment modalities and evaluate subsequent reproductive outcomes. **Methods**: A systematic search of Web of Science, Scopus, and PubMed identified studies published up to January 2026. The review followed PRISMA guidelines and SWiM reporting standards. Eligible studies included case reports, randomized controlled trials, prospective and retrospective cohort studies, and case series published in English. Only studies reporting women desiring future conception and specifying treatment type for recurrent CSP were included. Comparative analyses were restricted to treatment groups with at least 40 patients. Recurrence rates among five treatment groups were compared using the two-proportion z-test. **Results**: Overall, 1991 women treated for CSP were included. Of these, 636 (32%) desired future pregnancy, and 453 (71.1%) subsequently conceived. Fifty-four recurrent CSPs were reported, corresponding to a recurrence rate of 11.9%. Higher recurrence rates were observed after dilation and curettage with or without uterine artery embolization than after hysteroscopic treatment. Among women achieving a subsequent pregnancy, 60.3% resulted in viable intrauterine pregnancies, 27.6% were non-ongoing pregnancies, and 11.9% represented recurrent CSP. **Conclusions**: Among the treatment groups analyzed, hysteroscopic management was associated with lower observed recurrence rates compared with dilation and curettage-based approaches. However, these findings should be interpreted cautiously, as treatment allocation was not randomized and substantial heterogeneity in CSP characteristics, patient selection, and follow-up duration may have influenced the observed differences. Prospective comparative studies are required to determine whether these differences reflect true treatment effects.

## 1. Introduction

Ectopic pregnancy (EP) is defined by the implantation of the blastocyst outside the uterine cavity. EP represents around 0.5–1% of all pregnancies and is the leading cause of maternal mortality during the first trimester. Around 95% of ectopic pregnancies implant in the fallopian tube, being defined as tubal pregnancy (TP). Non-tubal pregnancies (NTP) make up less than 5% of all EPs [1,2].

Caesarean scar pregnancy (CSP) is a non-tubal ectopic pregnancy representing one of the most severe complications following caesarean delivery, with an incidence estimated at 1 in 2200 pregnancies [3,4].

CSP arises from the implantation of the gestational sac within the area of the previous caesarean section (CS) scar, a condition that can lead to life-threatening complications such as severe haemorrhage, uterine rupture, and, in some cases, hysterectomy [5].

Early prenatal diagnosis of CSP is of paramount importance, as it facilitates the formulation of a pre-planned treatment approach in specialized centres with expertise in managing such complex anomalies. The diagnosis of CSP is commonly made via ultrasound, where the gestational sac is observed within the CS scar area, typically in the presence of an empty uterine cavity and a thinned myometrial layer [6,7,8].

Vial et al. were the first to propose, in 2000, a classification system distinguishing two distinct types of cesarean scar pregnancies (CSPs) [9].

Type I, or ‘on-the-scar’ CSP, refers to implantation of the gestational sac on the cesarean scar, with growth directed toward the cervico-isthmic space and the uterine cavity.

Type II, or ‘in-the-niche’ CSP, involves implantation deep within the scar defect, often leading to extensive invasion toward the bladder and abdominal cavity [9].

The clinical course of CSP is highly unpredictable and may result in potentially fatal complications, including uterine rupture and haemorrhage, during the early stages of pregnancy. Management of CSP remains challenging, as no universally accepted treatment algorithm has been established. Available options include expectant management, methotrexate-based therapies, uterine artery embolization, dilation and curettage, hysteroscopic treatment, and laparoscopic or laparotomic surgical resection. Treatment selection depends on CSP characteristics, gestational age, haemodynamic status, residual myometrial thickness, and local expertise. Although dilation and curettage-based approaches remain widely used due to their accessibility, concerns regarding incomplete treatment and haemorrhagic complications have increased interest in hysteroscopic and laparoscopic techniques. However, comparative evidence remains limited, and the optimal strategy for preserving fertility and reducing recurrence risk is still uncertain.

Furthermore, there is a notable lack of consistent data regarding the reproductive outcomes following CSP. Existing studies are often limited by small sample sizes, variability in treatment modalities, and discrepancies in the outcomes investigated, which impedes the ability to draw firm conclusions regarding the true risk of adverse outcomes in women with a history of CSP [10,11].

Considering the lack of consensus guidelines in terms of best treatment-management, the primary objective of this literature review is to compare the recurrence rates of CSP among current treatment options and to explore whether the type of treatment selected for CSP is associated with the recurrence of scar pregnancies, with the aim of establishing the most effective treatment approach. As a secondary objective, we analyzed outcomes of intrauterine pregnancies—live birth (term and preterm), PAS, and severe complications.

## 2. Materials and Methods

### 2.1. Search Strategy

**Figure 1:**
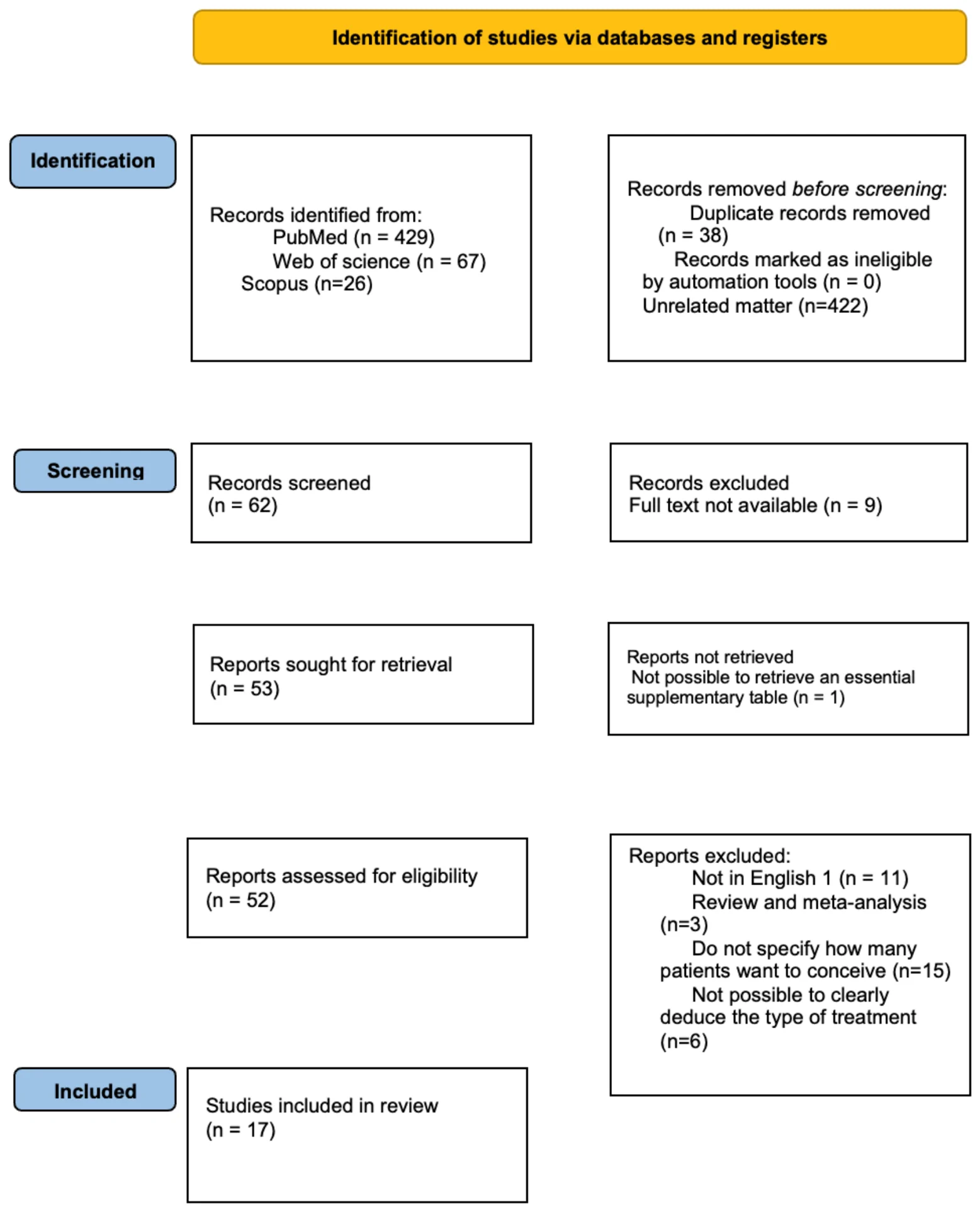
PRISMA flow diagram.

This systematic review was conducted in accordance with the PRISMA guidelines for systematic reviews and the Synthesis without Meta-analysis (SWiM) Guidelines (see Supplementary Tables S1 and S2) [12]. Two independent reviewers (L.V. and A.C.) performed a comprehensive literature search of the Web of Science, Scopus, and PubMed databases, including all studies published up to January 2026, without any date restrictions. The search strategy combined the following keywords and MeSH terms: (“cesarean scar pregnancy” OR “CSP”) AND (recurrence OR recurrent) AND (treatment OR management). Only studies that specified the number of patients with a declared desire to conceive were included. Recurrence rates were then analysed according to the type of treatment for caesarean scar pregnancy (CSP). The study selection process is detailed in the PRISMA flow diagram (Figure 1).

### 2.2. Eligibility Criteria

Eligible study designs included case reports, randomized controlled trials, prospective controlled studies, prospective cohort studies, retrospective studies, and case series. Only full-text articles published in English were included. However, after full-text screening, only case series met the inclusion criteria. Systematic reviews, meta-analyses, letters to the editor, and conference abstracts were excluded. However, reference lists of relevant reviews were manually screened to identify additional eligible studies. Studies with unclear, incomplete, or low-quality data, or those reporting non-quantifiable outcomes, were excluded. Studies that did not specify the number of patients desiring future conception out of the total number of treated patients were excluded. We also excluded studies that did not specify the type of treatment received by patients who experienced a recurrence CSP. Among the outcomes of pregnancies following CSP treatment, we considered only the immediately subsequent pregnancy, excluding any descriptions of further subsequent pregnancies.

### 2.3. Data Extraction and Risk of Bias Assessment

All records identified through database searches were screened for publication year, citation details, title, authorship, abstract, and full text. Duplicate records were manually identified and removed independently by two reviewers (A.C. and T.L.). Titles and abstracts of the remaining articles were independently screened by the same reviewers to exclude irrelevant studies. Full texts of potentially eligible studies were then independently assessed for inclusion. Discrepancies were resolved by discussion and consensus. The methodological quality of included studies was assessed using the JBI Critical Appraisal Checklist for Case Series (Supplementary Table S3). This study has been registered in the PROSPERO database (Registration number: CRD420251113275). After the screening process, all included studies were case series.

### 2.4. Data Synthesis and Statistical Analysis

Data extracted included the total number of patients treated in each study, the number of patients desiring future conception, the type of treatment received, the follow-up period, and the number of subsequent pregnancies. We specified the number of intrauterine normal pregnancies, non-viable pregnancies, and recurrent cesarean scar pregnancies. Additionally, we investigated the outcomes of intrauterine pregnancies and recurrent scar pregnancies that were managed expectantly.

The study included fifteen treatment groups undergoing different treatment approaches, with highly variable sample sizes. Because recurrence estimates derived from very small groups may be unstable and associated with substantial uncertainty, the primary comparative analysis was restricted to treatment groups including ≥40 patients. This threshold was selected as a pragmatic criterion to allow comparison among treatment groups with sufficient available data, rather than as a validated methodological cutoff. Statistical significance of differences in recurrence rates among treatment groups was assessed using pairwise two-proportion z-tests (SPSS version 20, IBM, Armonk, NY, USA). A *p*-value < 0.05 was considered statistically significant. Ninety-five percent confidence intervals for differences in proportions were calculated using the Newcombe method for independent samples.

To evaluate the robustness of the findings, sensitivity analyses were performed using alternative sample size thresholds (≥30 and ≥50 patients).

Treatment groups may differ systematically in baseline characteristics, including CSP type, severity, gestational age at treatment, and criteria guiding treatment selection.

Given the observational nature of the available evidence, these comparisons were considered exploratory and were not intended to establish causal relationships between treatment modality and recurrence risk and no formal pooled meta-analysis was performed; therefore, the findings should be interpreted with caution.

## 3. Results

We identified 522 manuscripts, of these 429 were identified on PubMed, 67 were identified on Web of Science, 26 on Scopus. Records excluded for selection criteria and duplicates were *n* = 460. We included in our review a total of seventeen manuscripts at the end of the screening process [13,14,15,16,17,18,19,20,21,22,23,24,25,26,27,28,29]. The PRISMA flow diagram of the selection process is provided in Figure 1.

The dates of all manuscripts analyzed are summarised in Table 1. Out of a total of 1991 patients treated in the studies included in this review, 636 expressed a desire for future pregnancy (32%) and 453 achieved a subsequent pregnancy (71.13%). Of the 453 pregnancies achieved, we report 54 recurrences, 11.9%. To address our primary outcome, we grouped the patients who desired a future pregnancy according to the type of treatment they had undergone for the first episode of CSP, and evaluated the recurrence rate observed in each group (Table 2). To enhance clarity, we summarized the results by ordering the groups, decreasing the number of patients treated per approach. From the total number of patients desiring to conceive, we excluded the 16 patients reported by Şeyhanlı Z et al. [27], as the authors did not specify the treatment received by the patient who achieved a subsequent pregnancy.

From our search, 15 different principal treatment approaches have been adopted for the management of CSP. Xinyi Sun et al. [21] did not specify the type of adjuvant treatment used in the 10 patients treated with D&C; for this reason, these patients were excluded in statistical analysis from the total patients treated with D&C.

**Figure 2:**
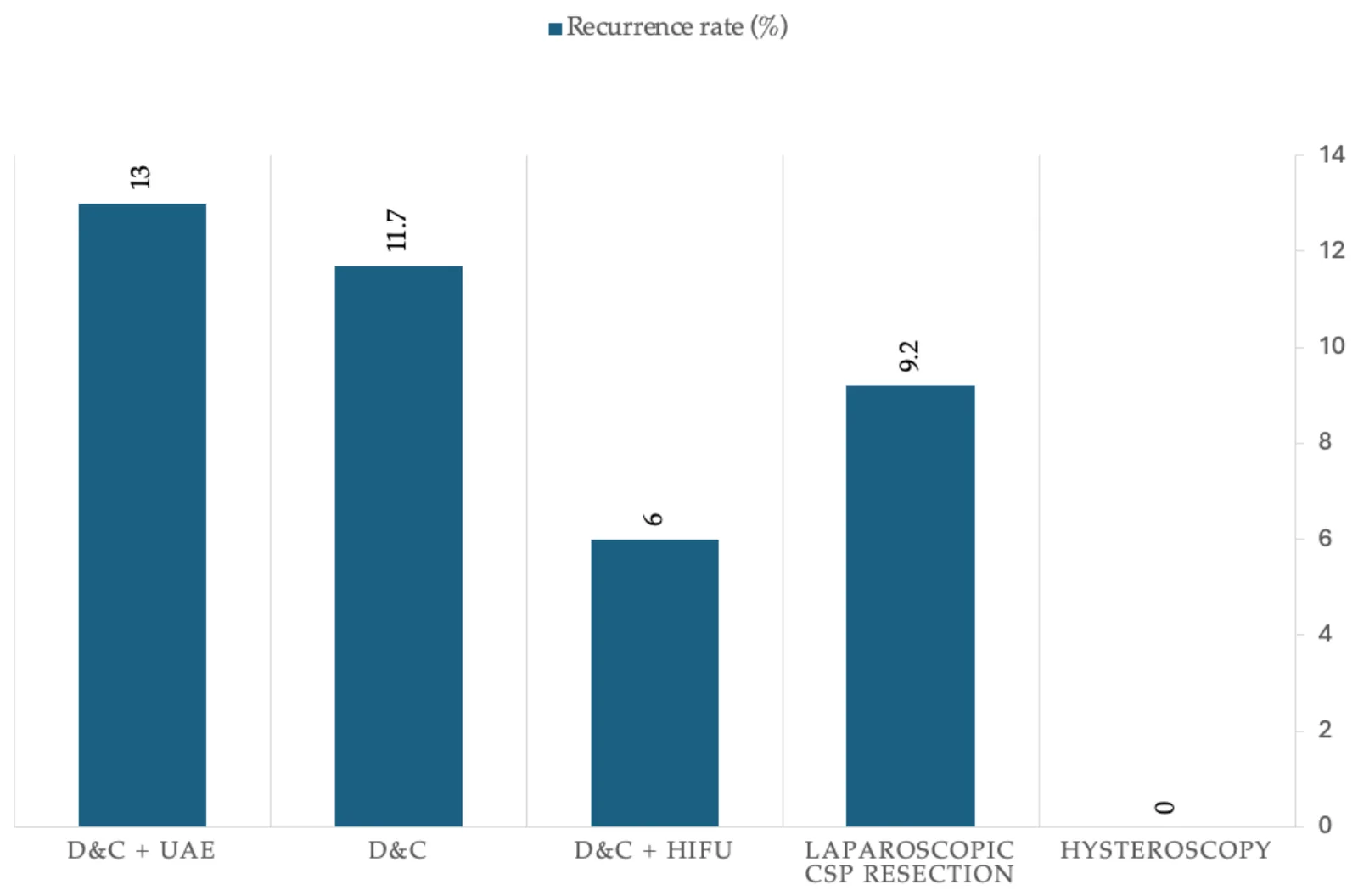
Recurrence rates of recurrent caesarean scar pregnancy according to treatment modality.

For 10 treatment strategies, the sample size was < 40 patients and, as shown, 4 of these treatments had a 0% recurrence rate. We compared the recurrence rates across the five patient groups with ≥ 40 patients: Group 1 (13% out of 154 patients), Group 2 (11.7% out of 128 patients), Group 3 (6% out of 100 patients), Group 4 (9.2% out of 54 patients), and Group 5 (0% out of 40 patients). The distribution of recurrence rates among the five treatment groups included in the comparative analysis is graphically summarized in Figure 2.

The analysis of differences in proportions among the five groups was performed using two-tailed z-tests for pairwise comparisons, together with 95% confidence intervals for differences in proportions (Table 3). The five treatment groups included in the comparative analysis are defined in the Table 3 footnote.

The contrast between Group 1 and Group 5 showed the largest difference (13.0%, 95% CI 3.8–24.5; z = 2.407; *p* ≈ 0.016), indicating a statistically significant difference in recurrence rates. Similarly, Group 2 vs. Group 5 (11.7%, 95% CI 2.3–23.7; z ≈ 2.269; *p* ≈ 0.023) also demonstrated a significant difference, whereas Group 4 vs. Group 5 (9.2%, 95% CI −0.4–19.6; z ≈ 1.978; *p* ≈ 0.048) showed a borderline difference with the lower bound of the confidence interval crossing zero. These findings suggest heterogeneity in recurrence rates across treatment strategies, although, except for comparisons involving the zero-event group (Group 5), incidence rates among Groups 1–4 do not differ substantially from each other.

Sensitivity analyses were performed to assess the impact of the sample size threshold used for treatment group selection. When treatment groups including ≥30 patients were considered, an additional treatment group (D&C combined with laparoscopic management, *n* = 34) was included. The overall interpretation of the results remained unchanged, with recurrence patterns comparable to the primary analysis and no additional statistically significant differences emerging. Conversely, when applying a more restrictive threshold of ≥50 patients, the hysteroscopic treatment group (*n* = 40) was excluded, resulting in the loss of one clinically relevant treatment category. Under this stricter threshold, no statistically significant differences among the remaining treatment groups were observed, suggesting that the comparative findings should be interpreted cautiously and within the context of available evidence.

As a secondary objective, we analyzed the outcomes of 453 intrauterine—normal located—pregnancies. While Carry Verberkt et al. [24] provided information on the number of patients with recurrent CSP and the treatments they underwent, the authors did not report the number of successful intrauterine pregnancies or non-ongoing pregnancies. Therefore, excluding their 32 subsequent pregnancies, among the remaining 421 cases, 254 resulted in a live birth (60.33%), and 116 were non-ongoing pregnancies (27.55%).

Of the 254 patients with a normal intrauterine pregnancy, gestational age was specified in 199 cases (Table 4). In 55 patients, this information was either missing or incomplete. Eight patients had abnormal placentation (4%), and four underwent postpartum hysterectomy due to related complications (2%). Among the patients with recurrent cesarean scar pregnancy (CSP), two chose expectant management. One of them developed placenta previa and underwent cesarean section at 36 + 1 weeks, followed by a subtotal hysterectomy due to severe postpartum hemorrhage.

## 4. Discussion

CSP has emerged as a growing concern in modern obstetrics and gynecology, with a noticeable increase in incidence over the past years. [13]. The rates of subsequent pregnancies in women with a history of CSP and a desire for future conception are largely consistent with those reported in other recently published studies, being 71.13% in our review vs. 70% reported by Morlando et al. [31]. In contrast, our analysis revealed a CSP recurrence rate of 11.9%, which is lower than the 18% reported in other studies [13].

The best therapeutic approach is still a matter of debate, as suggested by the results of the present study, in which 15 types of treatment (single or combined) were adopted, and for 10 of them, the patient sample is <40 patients.

Given the absence of a universally accepted minimum sample size threshold for observational studies evaluating CSP recurrence, the cutoff of ≥40 patients was selected pragmatically to reduce the instability of recurrence estimates derived from very small treatment groups. This criterion was not intended as a validated methodological threshold, but rather as an exploratory approach to allow more reliable comparisons among treatment strategies. Small treatment groups, particularly those reporting zero recurrences, may provide imprecise estimates because the absence of observed events may reflect limited sample size, insufficient follow-up, or under-reporting rather than a true absence of recurrence. Therefore, these groups were not included in the primary comparative analysis.

Focusing on the five treatment options with a sample size of ≥40 patients each, we performed an exploratory comparison of recurrence rates across different treatment strategies.

Among the five treatment groups analyzed, Groups 1 and 2 demonstrated the highest recurrence rates (13% and 11.7%, respectively) when compared to Groups 3 (6%) and 5 (0%). These findings suggest that treatments associated with Groups 1 and 2 may be less effective in reducing recurrence risk. However, these differences should not be interpreted as evidence of treatment superiority, since all included studies were case series and treatment allocation was not randomized. Selection bias, together with heterogeneity in CSP severity, gestational age at treatment, scar characteristics, and operator experience, may have substantially influenced both treatment choice and subsequent outcomes.

Group 5, despite reporting a 0% recurrence rate, includes only 40 patients. Although this outcome is encouraging and may indicate a potentially effective treatment approach, the small sample size and resulting limited statistical power prevent definitive conclusions. It is also possible that the absence of recurrence reflects insufficient follow-up duration or under-sampling, rather than true efficacy.

Therefore, while Groups 1 and 2 appear to be associated with poorer outcomes, the treatment used in Group 5 could represent a promising option that warrants further investigation. Larger prospective studies with adequate follow-up are necessary to validate these preliminary findings and better determine the relative effectiveness of the treatments.

A relevant mechanistic consideration is that differences in recurrence rates may also be influenced by the extent to which each treatment addresses the underlying cesarean scar defect. Surgical approaches such as laparoscopic or laparotomic CSP resection aim to directly excise and repair the myometrial defect, whereas dilation and curettage primarily removes gestational tissue without restoring the structural integrity of the uterine wall. Therefore, observed differences in outcomes across treatment groups may reflect differences in defect correction rather than the procedural approach itself.

It should also be acknowledged that CSP was not stratified according to Vial’s classification (Type I vs. Type II), nor were key anatomical and clinical variables such as niche depth, residual myometrial thickness, or gestational age consistently reported in the included studies [9]. Therefore, these factors could not be systematically analyzed. Their absence in a substantial proportion of the available literature limits the ability to fully adjust for clinically relevant confounders and may have influenced both treatment selection and outcomes.

The secondary objective shows that among the 421 subsequent pregnancies with full data available, 254 resulted in a live birth (60.33%), and 116 were non-ongoing pregnancies (27.55%).

Therefore, we can conclude that among the 32% of women desiring pregnancy after CSP treatment, approximately 60% will achieve a normally progressing intrauterine pregnancy, about 30% will experience a non-viable pregnancy (miscarriage or ectopic pregnancy), and around 12% will experience a recurrence.

## 5. Limitations and Bias

We are aware that the relatively small sample sizes in certain groups, particularly Group 5, may have limited the statistical power to detect true differences. Since all the reviewed studies were case series, which offer lower-quality evidence than randomized trials or large cohort studies, the risk of bias is higher, and the results may not be widely generalizable. An important limitation is the substantial heterogeneity among the included studies, particularly regarding patient populations, follow-up duration, CSP classification, gestational age at treatment, and surgeon expertise. Since comparative analyses were conducted using aggregated study-level data derived from heterogeneous case series, the reported differences between treatment groups should be interpreted cautiously. An additional methodological limitation is related to the selection of the minimum sample size threshold for comparative analyses. Since no validated cutoff exists for this type of observational evidence, the ≥40-patient criterion was selected pragmatically. Although sensitivity analysis using a ≥30-patient threshold showed comparable results, analyses restricted to groups with ≥50 patients were limited by the exclusion of clinically relevant treatment categories and did not demonstrate statistically significant differences. Therefore, the reported comparisons should be considered exploratory rather than definitive estimates of comparative treatment efficacy.

Another source of bias is the time span of the study. Although 11 years may seem relatively short in many medical fields, in the case of hysteroscopy it represents a longer period, as its use and the level of expertise are not evenly distributed worldwide. This contrasts with more widely practiced surgical techniques such as dilation and curettage (D&C), which are commonly used across different settings. An additional limitation of this study is the inability to account for variations in gestational age at the time of CSP treatment, as well as the specific CSP type (Type I or Type II), due to the absence of these data in a substantial proportion of the manuscripts reviewed. A major limitation of this study is that the follow-up duration varied substantially across the included studies, ranging from 24 months to not reported, introducing potential time-related bias. Since recurrence is a time-dependent outcome, shorter or unreported follow-up may lead to underestimation of true recurrence rates. For these reasons, the reported crude proportions should be interpreted as exploratory associations rather than causal estimates of treatment effect.

The restriction to English-language publications may have introduced language bias, as potentially relevant studies published in other languages may have been excluded. However, this criterion was applied consistently during study selection.

## 6. Conclusions

Hysteroscopy, when performed by experienced clinicians, is progressively emerging as a promising option in the management of CSP. This review suggests that traditional D&C, although widely used, may be associated with higher recurrence rates. Considering the already reduced reproductive potential of patients with previous obstetric surgery, minimally invasive approaches with lower surgical impact may represent valuable alternatives, particularly in specialized hysteroscopic centres. We are aware of the limitations and potential biases of our results, particularly due to the small sample size and retrospective nature of the available studies. Future prospective studies with larger and more balanced cohorts are warranted to confirm these findings and to further investigate potential clinical and biological factors that may influence reproductive outcomes and recurrence patterns. We believe that the data presented may serve as a valuable resource for clinicians, supporting therapeutic decision-making and providing objective data to guide patient counselling.

## Figures and Tables

**Table 1:** Characteristics and Extracted Data of Included Studies.

Author, Year	Total Patients Treated	Patients Who Want to Conceive	Treatment	Minimum Follow-Up Period (Months)	Subsequent Pregnancies (*n*)	Normal Implanted Intrauterine Pregnancies	Non-Ongoing Pregnancies	Recurrent CSP
Yamaguchi M., 2014 [14]	8	5	5 Local MTX ^1^	60	5	3	1 Miscarriage	1
Gao L., 2016 [15]	28	8	4 D&C ^2^ + MTX systemic 1 D&C + UAE ^3^ 1 MTX + UAE + D&C + LPT ^4^ repair 2 D&C + LPT repair	24	7	2 D&C + MTX 1 D&C + UAE 1 MTX + UAE + D&C + LPT repair	2 Miscarriage 1 D&C + MTX 1 D&C + LPT repair	1 D&C + MTX
Orhan A., 2019 [16]	30	14	4 D&C 3 Systemic MTX 1 D&C + Systemic MTX 1 Expectant management 4 Combined MTX 1 Local MTX	NA	9	6 (of which 1 is an ongoing pregnancy at the time of paper’s writing)	0	3 D&C
Stępniak A., 2019 [17]	22	10	D&C+ Systemic MTX	9	6	5	1 Miscarriage	0
Zhang C., 2019 [18]	154	28	USg ^5^-D&C	18	23	13 intrauterine pregnancy (of which 1 is an ongoing pregnancy at the time of paper’s writing)	5 Induced abortion 3 Tubal ectopic pregnancies	2
Xu X. 2020 [19]	117	57	16 USg-D&C 34 LPS ^6^ monitored D&C 7 LPS CSP resection	12	9 USg-D&C 12 LPS monitored D&C 3 LPS CSP resection	8 USg-D&C 9 LPS monitored D&C 3 LPS CSP resection	1 Miscarriage—LPS monitored D&C 1 Tubal ectopic pregnancy—USg-D&C 1 Induced abortion for fetal malformation—LPS monitored D&C	1 LPS monitored D&C (Expectant management)
Lou T., 2020 [20]	53	10	MTX + UAE + D&C	NA	8	6	/	2
Sun X., 2021 [21]	149	51	10 D&C +/− not specified adjuvant therapy 39 HSC ^7^ 2 LPS CSP resection	24	44	38	6 Miscarriage	0
Hofgaard E., 2021 [22]	14	14	Robot-assisted LPS pregnancy removal and scar repair	6	9	8	0	1
Chen Y.T., 2022 [23]	53	23	D&C + UAE	NA	19	15	3 Miscarriage	1
Verberkt C., 2023 [24]	60	37	1 Expectant management 1 HSC 3 Local MTX 4 Systemic MTX 1 Combined MTX 17 USg-D&C 10 LPS CSP resection	48	32	NA	NA	1 USg-D&C 1 Expectant management
Jin X., 2023 [25]	499	51	3 Local MTX 15 D&C + UAE 2 USg-D&C 17 USg local lauromacrogol injection + D&C	NA	37	31	2 Tubal pregnancy	2 D&C + UAE 1 USg-D&C 1 Lauromacrogol + D&C
Wang X., 2023 [26]	272	192	100 HIFU ^8^ + USg-D&C 92 UAE + USg-D&C	30	78 HIFU + USg-D&C 66 UAE + USg-D&C	27 HIFU + USg-D&C (3 ongoing pregnancies at the time of the paper’s writing) 21 UAE + USg-D&C (2 ongoing pregnancies at the time of the paper’s writing)	HIFU + D&C 2 Tubal pregnancies 2 Spontaneous abortion 41 Induced abortion UAE + D&C 1 Tubal pregnancy 1 Spontaneous abortion 30 Induced abortion	6 HIFU + D&C 13 UAE + D&C
Şeyhanlı Z., 2024 [27]	60	16	2 Systemic MTX 12 D&C+ Systemic MTX 41 D&C 2 LPT Wedge resection 2 LPT wedge resection + MTX 1 LPT wedge resection + MTX + D&C	NA	14	7	7 Miscarriage	0
Lei Y., 2024 [28]	379	100	61 USg-D&C 18 USg-D&C + UAE 21 LPS pregnancy removal and scar repair	60	62	41	6 Miscarriage	7 USg-D&C 4 USg-D&C + UAE 4 LPS pregnancy removal and scar repair
Ishikawa H., 2025 [29]	11	10	D&C with local injection of diluted vasopressin	NA	5	4	-	1
Feng Y., 2025 [30]	82	10	5 UAE + D&C 5 MTX + UAE + D&C	NA	5	4 (of which 1 is an ongoing pregnancy at the time of the paper’s writing)	0	1 MTX + UAE + D&C

^1^ Methotrexate; ^2^ Dilatation and Curettage; ^3^ Uterine Artery Embolization; ^4^ Laparotomic; ^5^ Ultrasound-guided; ^6^ Laparoscopic; ^7^ Hysteroscopy; ^8^ High-Intensity Focused Ultrasound. Footnotes. For studies reporting multiple treatment modalities within the same cohort, the number preceding each treatment indicates the number of patients who received that specific treatment. The reproductive outcomes reported in the subsequent columns refer exclusively to the corresponding treatment subgroup and not to the entire study population.

**Table 2:** Treatment-Specific CSP Recurrence Rates in Women Planning Future Pregnancy.

Treatment	Recurrence/Total	Rate (%)
D&C ^1^ + UAE ^2^ (Group 1) *	20/154	13%
D&C (Group 2) *	15/128	11.7%
D&C + HIFU ^3^ (Group 3) *	6/100	6%
LPS ^4^ CSP resection (Group 4) *	5/54	9.2%
HSC ^5^ (Group 5) *	0/40	0%
D&C + LPS	1/34	2.9%
D&C + Systemic MTX ^6^	1/15	6.7%
D&C + Macrogol	1/17	5.9%
D&C + Systemic MTX + UAE	3/15	20%
Local MTX	1/12	8.3%
Systemic MTX	0/7	0%
Combined MTX	0/5	0%
Expectant management	1/2	50%
D&C + LPT ^7^	0/2	0%
D&C + Systemic MTX + UAE + Laparotomic CSP resection	0/1	0%

* Five patient groups with ≥ 40 patients. ^1^ Dilatation and Curettage; ^2^ Uterine Artery Embolization; ^3^ High-Intensity Focused Ultrasound; ^4^ Laparoscopic; ^5^ Hysteroscopy; ^6^ Methotrexate; ^7^ Laparotomic.

**Table 3:** Comparison of CSP Recurrence Rates Across Patient Groups with ≥ 40 Individuals.

Treatment Group A	Treatment Group B	z-Stat	*p*-Value	Difference (%)	95% CI
Group 1	Group 2	0.322	0.748	1.3	(−5.1; 7.7)
Group 1	Group 3	1.795	0.0727	7.0	(−0.7; 14.7)
Group 1	Group 4	0.725	0.469	3.8	(−6.6; 14.2)
**Group 1**	**Group 5**	**2.407**	**0.0161**	**13.0**	**(3.8; 24.5)**
Group 2	Group 3	1.482	0.138	5.7	(−2.1; 13.5)
Group 2	Group 4	0.485	0.628	2.5	(−7.8; 12.8)
**Group 2**	**Group 5**	**2.269**	**0.0233**	**−3.2**	**(−11.4; 5.0)**
Group 3	Group 4	−0.794	0.454	11.7	(2.3; 23.7)
Group 3	Group 5	1.583	0.113	6.0	(−2.4; 14.8)
**Group 4**	**Group 5**	**1.978**	**0.0479**	**9.2**	**(−0.4; 19.6)**

Treatment group definitions: Group 1: D&C + UAE; Group 2: D&C; Group 3: D&C + HIFU; Group 4: Laparoscopic CSP resection; Group 5: HSC. Footnotes. Bold values indicate statistically significant results in between-group comparisons (*p* < 0.05).

**Table 4:** Normal implanted intrauterine pregnancies’ outcomes.

Normal Implanted Intrauterine Pregnancies with Complete Data (*n*)	At Term Delivery (*n*)	Preterm Delivery (*n*)	Abnormal Placental Implantation (*n*)	Hysterectomies (*n*)
199	154	45	6 Placenta Accreta Spectrum 2 Placenta previa	4

## Data Availability

The authors confirm that the data supporting the findings of this study are available within the article.

## Supplementary Materials

The following supporting information can be downloaded at: https://www.mdpi.com/article/10.3390/jcm15166239/s1, Table S1: PRISMA check list; Table S2: Synthesis Without Meta-analysis (SWiM) reporting items; Table S3: JBI Critical Appraisal Checklist for Case Series.

## Abbreviations

The following abbreviations are used in this manuscript:

CSP	Cesarean scar pregnancy
MTX	Methotrexate
D&C	Dilatation and Curettage
UAE	Uterine Artery Embolization
LPT	Laparotomy
LPS	Laparoscopy
HSC	Hysteroscopy
HIFU	High-Intensity Focused Ultrasound
