# Supplementary material for: Treatment-Based Recurrence of Scar Pregnancy: A Systematic Review of the Literature as a Tool for More Informed Decision-Making
Source: J Clin Med. 2026 Aug 12;15(16):6239. doi: 10.3390/jcm15166239 (PMC13513461; doi:10.3390/jcm15166239)
Supplement: Supplementary file 1 [file jcm-15-06239-s001.zip › Table S3. JBI Critical Appraisal Checklist for Case Series.pdf]

Title	Author, Year	D1	D2	D3	D4	D5	D6	D7	D8	D9	D10
Transvaginal methotrexate injection for the treatment of cesarean scar pregnancy: Efficacy and subsequent fecundity	Yamaguchi M., 2014	YES	YES	YES	YES	YES	YES	YES	YES	YES	YES
Reproductive outcomes following cesarean scar pregnancy – a case series and review of the literature	Gao L., 2016	YES	YES	YES	YES	YES	YES	YES	YES	YES	YES
Different treatment modalities and outcomes in cesarean scar pregnancy: a retrospective analysis of 31 cases in a university hospital	Orhan A., 2019	YES	YES	YES	YES	YES	YES	YES	YES	YES	YES
Effectiveness, complications and reproductive outcome of selective chemoembolization with methotrexate followed by suction curettage for caesarean scar pregnancy - A prospective observational study	Stępniak A., 2019	YES	YES	YES	YES	YES	YES	YES	YES	YES	YES
Outcomes of subsequent pregnancies in patients following treatment of cesarean scar pregnancy with high intensity focused ultrasound followed by ultrasound-guided dilation and curettage	Zhang C., 2019	YES	YES	YES	YES	YES	YES	YES	YES	YES	YES
Surgical outcomes of cesarean scar pregnancy: an 8-year experience at a single institution	Xu X. 2021	YES	YES	YES	YES	YES	YES	YES	YES	YES	YES
Reproductive outcomes of cesarean scar pregnancies pretreated with methotrexate and uterine artery embolization prior to curettage	Lou T., 2020	YES	YES	YES	YES	YES	YES	YES	YES	YES	YES
Subsequent Fertility in Women Treated for Caesarean Scar Pregnancy With Hysteroscopy: A 5-Year Follow-Up Descriptive Study in a Tertiary Hospital	Sun X., 2021	YES	YES	YES	YES	YES	YES	YES	YES	YES	YES
Cesarean scar pregnancy: Reproductive outcome after robotic laparoscopic removal with simultaneous repair of the uterine defect	Hofgaard E., 2021	YES	YES	YES	YES	YES	YES	YES	YES	YES	YES
Reproductive outcomes of cesarean scar pregnancies treated with uterine artery embolization combined with curettage	Chen Y.T., 2022	YES	YES	YES	YES	YES	YES	YES	YES	YES	YES
Effectiveness, complications, and reproductive outcomes after cesarean scar pregnancy management: a retrospective cohort study	Verberkt C., 2023	YES	UNCLEAR	YES	YES	YES	YES	UNCLEAR	UNCLEAR	YES	YES

Subsequent fertility after cesarean scar pregnancy: a retrospective analysis	Jin X., 2023	YES	YES	YES	YES	YES	YES	YES	YES	YES	YES
Clinical efficacy and re-pregnancy outcomes of patients with previous cesarean scar pregnancy treated with either high-intensity focused ultrasound or uterine artery embolization before ultrasound-guided dilatation and curettage: a retrospective cohort study	Wang X., 2023	YES	YES	YES	YES	YES	YES	YES	YES	YES	YES
Management and reproductive outcomes following cesarean scar pregnancy: experiences of two tertiary centers in a cohort of 60 women	Şeyhanlı Z., 2024	YES	UNCLEAR	YES	YES	YES	YES	UNCLEAR	UNCLEAR	UNCLEAR	YES
Surgical treatment and reproductive outcomes in caesarean scar pregnancy at a single center	Lei Y., 2024	YES	YES	YES	YES	YES	YES	YES	YES	YES	YES
Outcomes of local vasopressin injection and vaginal evacuation for cesarean scar pregnancy: A retrospective review of 11 cases	Ishikawa H., 2025	YES	YES	YES	YES	YES	YES	YES	YES	YES	YES
Uterine artery embolization plus ultrasound guided curettage for cesarean scar pregnancy: Five-year experience at a single tertiary hospital	Feng Y., 2025	YES	YES	YES	YES	YES	YES	YES	YES	YES	YES

Table S3: JBI Critical Appraisal Checklist for Case Series
